# Validation of a one-step reverse transcription PCR detection method for *Tobamovirus maculatessellati*, in tomato (*Solanum lycopersicum* L.) and pepper (*Capsicum annuum* L.)

**DOI:** 10.3389/fpls.2025.1535175

**Published:** 2025-01-29

**Authors:** Chellappan Padmanabhan, Andrea Gilliard, Kai-Shu Ling, Yazmín Rivera

**Affiliations:** ^1^ United States Department of Agriculture, Animal Plant Health Inspection Service, Plant Protection and Quarantine, Science and Technology, Plant Pathogen Confirmatory Diagnostics Laboratory, Laurel, MD, United States; ^2^ United States Department of Agriculture, Agricultural Research Service, U. S. Vegetable Laboratory, Charleston, SC, United States

**Keywords:** *Tobamovirus maculatessellati*, tomato mottle mosaic virus, RT-PCR, method validation, tomato, pepper

## Abstract

The solanaceous-infecting tobamoviruses are closely related and hence it can be challenging to detect them using serological or molecular methods, particularly when present in a mixed infection. Tomato mottle mosaic virus (ToMMV) is a newly identified tobamovirus that poses serious risk to tomato (*Solanum lycopersicum* L.) and pepper (*Capsicum annuum* L.) production worldwide. Species-specific identification is crucial to prevent the entry and establishment of plant pathogens and protect the billion-dollar tomato industry. In this study, we report the validation of a previously described reverse transcription polymerase chain reaction (RT-PCR) assay that amplifies a 289 bp fragment of the coat protein coding region of ToMMV genome. This assay has 100% specificity for ToMMV. Inclusivity tests were performed against a diverse collection of six ToMMV isolates in North America. Exclusivity tests showed no cross reaction with eleven non-target viruses and seven viroids commonly found on tomato and pepper host plants. The detection limit of the one-step RT-PCR was determined to be at 10^-5^ (or 0.25pg/μl) dilution in plant samples, with its amplicon sequence confirmed by Sanger sequencing. The RT-PCR can detect ToMMV consistently on contaminated seed or leaf tissues. This validated assay could serve as a standard method for detecting ToMMV in seed health testing and for plant disease diagnosis, thus to prevent inadvertent introduction and spread of this emerging and economically important tobamovirus in tomato and pepper fields.

## 1 Introduction

Tomato (*Solanum lycopersicum* L.) is a globally important crop consumed by billions of people and it is cultivated in about 6.5 million hectares with annual production of 262.5 million tons produced worldwide, mainly from China, India, Turkey and United States (FAOSTAT, 2023). Tomato is rich in carotenoids, fiber content, minerals, vitamins, and flavonoids with limited caloric supply, and thus benefits human physiology and nutrition (Dorais et al., 2008).

Among the viruses infecting tomato and pepper, tobamoviruses are the most devastating (Hanssen et al., 2010). Tobamoviruses comprise 37 species that are not vector-transmissible and have a thermal inactivation point of 90°C, enabling their survival in plant and soil for many years (ICTV Report, 2024). Viral particles of tobamoviruses have a predominant length of 300–310 nm, 18 nm in diameter, with an undivided genome of 6.3–6.6 Kb. The single-stranded RNA genome encodes at least four proteins: two replication proteins of approximately 124-132 kDa and 181-189 kDa, the 28-31 kDa movement protein (MP) and the 17-18 kDa coat protein (Palukaitis et al., 2024). Tobacco mosaic virus (TMV) virus assembly operates in the cytoplasm while viral movement protein probably mediates virion cell-to-cell transfer (Ashby et al., 2006).

Based on genome sequences, serological specificity and host range, tobamoviruses are classified into at least nine subgroups, including those infecting the families Solanaceae, Brassicaceae, Cactaceae, Apocynaceae, Cucurbitaceae, Malvaceae, Leguminosae, Passifloraceae and Orchidaceae (Dorokhov et al., 2018; Li et al., 2017). Tomato mosaic virus (ToMV) and tobacco mosaic virus (TMV) belong to the subgroup I and are important members of the genus *Tobamovirus* due to their impact. ToMV is one of the most destructive viral diseases of tomato due to the virus stability, transmission efficiency and high rate of infectivity (López-Gresa et al., 2012). In 2013, a new Tobamovirus species was identified in tomato from Mexico, named Tomato mottle mosaic virus (ToMMV) or *Tobamovirus maculatessellati* that belongs to this subgroup I (Li et al., 2013). This new virus produced necrosis on the upper leaves of tomato seedlings, and mosaic patterns and deformation on leaves of mature plants (Li et al., 2013). After the first description, ToMMV has been detected in several states in the United States, including California, Florida, New York and South Carolina (Webster et al., 2014; Padmanabhan et al., 2015; Sui et al., 2017). According to the EPPO records, ToMMV is distributed worldwide (EPPO Global Database, 2024.), including Africa (Maudarbaccus et al., 2021), Asia (Che et al., 2018; Li et al., 2014, 2020; Luria et al., 2017; Turina et al., 2016); Europe (Ambrós et al., 2017; Fowkes et al., 2022; Schoen et al., 2023); North America (Li et al., 2013; Webster et al., 2014; Padmanabhan et al., 2015; Sui et al., 2017), and South America (Moreira et al., 2003; Nagai et al., 2018, 2019). Although it is not officially present in Australia, ToMMV was intercepted on imported pepper seeds (Lovelock et al., 2020). In addition, the Netherlands intercepted ToMMV in tomato seed imported from Asia (Fowkes et al., 2022). The presence of ToMMV in Brazil, dating back to 1992 (Moreira et al., 2003), has been recently confirmed based on the complete genome sequence (GenBank MH128145) of a tomato isolate (Nagai et al., 2018). The phylogenetic analysis revealed that the newly identified ToMMV is closely related to ToMV and TMV (Li et al., 2017). Currently, ToMMV is, along with ToMV and tomato brown rugose fruit virus (ToBRFV), a major threat to tomato crop productions (Carr, 2024). With the addition of ToMMV, the subgroup I of tobamoviruses includes ToMMV, ToMV, TMV, ToBRFV and Rehmannia mosaic virus (RheMV) (Adams et al., 2017). In addition to tomato, ToMMV might pose a serious risk to pepper (*C. annuum*) crops (Li et al., 2014; Lovelock et al., 2020).

Tobamovirus management in tomato has been accomplished by the use of cultivars with the resistance genes, including *Tm-1*, *Tm-2*, and *Tm-2^2^* genes in the last 60 years (Carr, 2024). The emergence of ToBRFV breaks the *Tm-2^2^* gene (Luria et al., 2017). However, the *Tm-2^2^* gene in tomato cultivars largely confers resistance to ToMMV infection, but this could be overcome in other cultivars with heterozygosity on the *Tm-2^2^* gene and under high temperature conditions (Nagai et al., 2019; Sui et al., 2017; Tettey et al., 2022, 2023). According to the California Department of Food and Agriculture (CDFA), there have been no reports of quantitative crop yield losses directly attributed to Tomato mottle mosaic Virus (ToMMV). However, it is anticipated that the losses could be similar to those caused by the closely related Tomato Mosaic Virus, which can lead to yield reductions of up to 25% in infected, non-resistant greenhouse or field-grown susceptible tomato crops (CABI, 2015). Therefore, strategies for detection and identification are essential to disease management programs and for successful eradication.

ToMMV-infected tomato plants are stunted, with severe leaf mosaic, mottling and leaf distortion symptoms that are difficult to distinguish from those infected by other tobamoviruses, such as TMV, ToMV and ToBRFV on tomato and pepper. In recent years, several detection methods have been developed for ToMMV detection, including multiplex RT-PCR (Sui et al., 2017; Tettey et al., 2022), real-time RT-PCR (Haghi et al., 2023; Tiberini et al., 2022), and Loop-mediated isothermal amplification assay (Kimura et al., 2023). Earlier developed a multiplex immunocapture RT-PCR assay for detection of tobamoviruses (Jacobi et al. 1998). For quarantine and regulatory purposes, it is important to develop a standard method that has been extensively validated on their sensitivity, specificity and repeatability to achieve a species-specific detection and identification for ToMMV. The validation of a diagnostic test provides the user and stakeholders with the confidence that its performance is adequate for its intended purpose (Groth-Helms et al., 2023). In the present study, we focused on validating a previously developed multiplex RT-PCR (Sui et al., 2017) for species-specific detection of ToMMV in a single-step RT-PCR reaction.

## 2 Materials and methods

### 2.1 Plant materials and virus isolates

ToMMV isolates used in the present study were originally collected by collaborator (Ling) at the USDA-ARS, U.S. Vegetable Laboratory from tomato plants grown in field or greenhouse in various states in the U.S. and Mexico, including South Carolina isolate V13-478 on a unknown cultivar (SC13-05, GenBank accession no. KX898033), New York isolate 1 V13-481 on a germplasm LA2109 (NY13-01, GenBank Accession No. KT810183), New York isolate 2 V13-481 (NY13-02), California isolate 1 V16-07 on a unknown cultivar (CA16-01, Genbank Accession No. KX898034), California isolate 2 V16-08 on a unknown cultivar (CA16-02) and ToMMV Mexican isolate -MX5 from a greenhouse with unknown cultivar (GenBank Accession No. KF477193). The California isolate of ToMMV (V16-07) was the primary virus isolate used for evaluation of single-step RT-PCR. However, all six ToMMV isolates listed above were used for validation assay. The non-target viruses included tomato spotted wilt virus (TSWV) and tomato ringspot virus (ToRSV) (from Dr. Tzanetakis, University of Arkansas), ToMV-PV0135, ToBRFV-PV1236, tomato chlorosis virus (ToCV)-PC1242, tomato infectious chlorosis virus (TICV)-PV1108, pepper mild mottle virus (PMMoV)-PV0165, and bell pepper mottle virus (BPeMV)-PC0170 positive samples purchased from North Rhine-Westphalia, Germany (DSMZ). TMV was purchased from Agdia (Elkhart, IN, USA). Seven positive viroid samples, including citrus exocortis viroid (CEVd), tomato chlorotic dwarf viroid (TCDVd), tomato apical stunt viroid (TASVd), Mexican papita viroid (MPVd), Columnea latent viroid (CLVd), tomato planta macho viroid (TPMVd), and potato spindle tuber viroid (PSTVd), used in this study were provided by the USDA-APHIS Plant Pathogen Confirmatory Diagnostics Laboratory (PPCDL). For sample preparation, the targeted and non-targeted virus samples were excised and chopped into small pieces in a biological safety cabinet. For RNA extraction, 100 mg of symptomatic tissue were transferred to 2 ml microcentrifuge tubes and stored at -80°C until further use.

### 2.2 RNA extraction

Total RNA was extracted using 100 mg infected and healthy leaf tissues of tomato and pepper. The tissue was homogenized using a FastPrep FP-24 instrument (MP Biomedical, CA, USA). The RNeasy Plant Mini Kit (Qiagen, USA) was used for total RNA extraction. Extracted RNA was treated with 1 unit of RNase free DNase I (Thermo Fisher Scientific, USA) per 1-5 μg of total RNA in a 50 μl total volume and incubated for 20-30 minutes at room temperature (25-37°C). The DNase-I treated RNA was resuspended in nuclease free water. RNA concentrations were measured with Qubit fluorometer (Invitrogen, Carlsbad, CA), and the quality was tested using TapeStation (Agilent, Santa Clara, CA) as per manufacturer’s instructions. For general testing, total plant RNA was extracted from tomato leaf tissue using a TRIzol reagent following the manufacturer’s instructions (Thermal Fisher Scientific, USA).

### 2.3 RT-PCR primers, reaction, and thermal cycling

**Figure 1:**
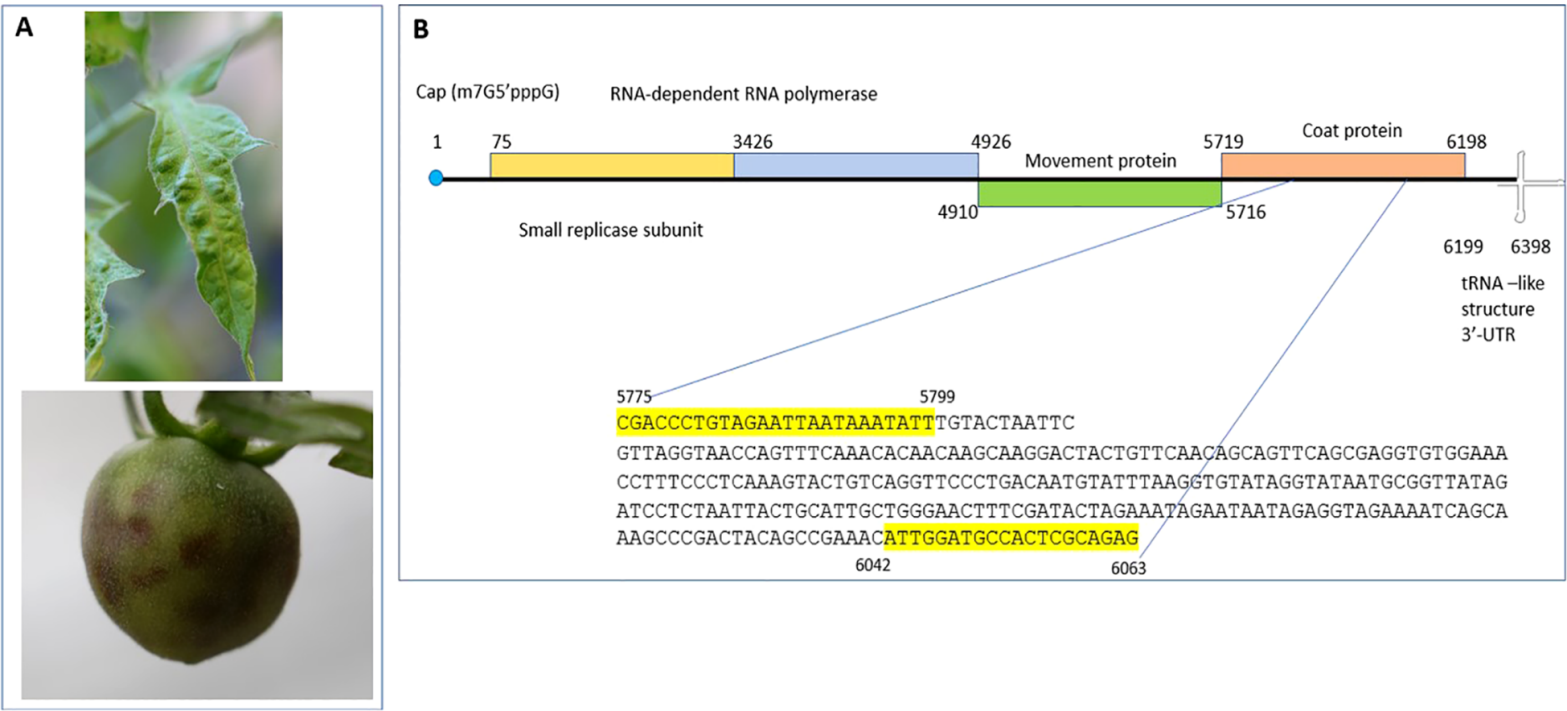
Tomato mottle mosaic virus infection on tomato plants and schematics of ToMMV genome organization. **(A)** Mottle mosaic symptoms of ToMMV infected tomato plants (leaf and fruit). **(B)** ToMMV genome organization with different open reading frames and location of RT-PCR primers and its amplicon sequence from ToMMV isolate V16-07.

To achieve species-specific detection of ToMMV, a set of specific primer pairs from Sui et al., 2017 were used. The specific forward primer ToMMV-F 5’- CGACCCTGTAGAATTAATAAATATT-3’ and reverse primer ToMMV-R 5’- CACTCTGCGAGTGGCATCCAAT-3’ were synthesized from IDT (Coralville, IA, USA) and were used in RT-PCR for method validation (**Figure 1**). Using a simplex RT-PCR assay capable of amplifying its respective expected viral amplicon of 289bp size, RT-PCR was performed using a Qiagen one-step RT-PCR kit (Qiagen Hilden, Germany).

A single RT-PCR reaction of 25 µl consisted of 5 µl of 5x Qiagen Onestep RT-PCR buffer, 1 µl Qiagen dNTPs (10 mM), 1 µl ToMMV forward primer (10 µM), 1 µl ToMMV reverse primer (10 µM), 1 µl Qiagen OneStep RT-PCR Enzyme Mix, 1 µl RNaseOUT (Invitrogen) (40U/µl), 13 µl Molecular Grade water, and 1 µl of each RNA template. Thermal cycling reaction was carried out on a Proflex (ThermoFisher Scientific, MA, USA), with reverse transcription at 50°C for 30 min, followed by 95°C for 15 min, and 35 cycles of 94°C for 1 min, 55°C for 45 sec, and 72°C for 1 min, with a final extension at 72°C for 10 min. RT-PCR products were analyzed using a TapeStation D1000 ScreenTape (Agilent, Santa Clara, CA) following manufacturer’s instructions or through electrophoresis on a 1.5% agarose gel containing 1:10,000 SYBR Safe DNA Gel Stain (Thermo Fisher Scientific, USA).

### 2.4 Evaluation of temperature and primer concentration tests

The RT-PCR was conducted to evaluate the melting temperature (Tm) of the target sequences. We tested a range of annealing temperatures, specifically 53°C, 55°C, 57°C, 59°C, and 61°C, to determine the optimal Tm for amplification. After assessing the amplification efficiency at these different annealing temperatures, we identified 55°C as the optimal Tm and selected it for further validation assays.

We tested different primer concentrations to optimize primer performance: 0.1 µM, 0.2 µM, and 0.4 µM. We assessed the effectiveness of these concentrations through amplification efficiency evaluations. The 0.4 µM primer concentration was determined to be the most effective and was selected for all subsequent validation experiments, including sensitivity, specificity, and selectivity assessments.

### 2.5 Sensitivity and specificity tests

Sensitivity was evaluated using 10-fold serial dilutions of total plant RNA from ToMMV-infected tomato plants, which were mixed with RNA from healthy plants, ranging from 10^0^ to 10^-8^. The amplicon at the limit of detection (10^-5^) was chosen for final identification through Sanger sequencing.

The specificity of the assay was analyzed using targeted ToMMV isolates (inclusivity) and with a non-target group (exclusivity) that included eleven viruses: TMV, ToMV, ToBRFV, TSWV, ToRSV, ToCV, TICV, PMMoV, BPeMV, WGMMV, and CGMMV. Additionally, seven viroids were also examined: CEVd, TCDVd, TASVd, MPVd, CLVd, TPMVd, and PSTVd. Positive and negative controls were included. The amplicons were processed using a TapeStation (Agilent, Santa Clara, CA) according to the manufacturer’s instructions.

### 2.6 Selectivity test

To evaluate the performance of RT-PCR under different tissue matrices, ToMMV leaf RNA was diluted to high (25 ng/μL, 10^-0^), medium (10^-2^), and low (10^-4^) concentrations and spiked in RNA extracted from various tissue matrices (tomato seeds, pepper seeds, and pepper leaf tissue) for RT-PCR testing. Consistent total plant RNA concentrations were used for all host matrices. The results of the selectivity assay were analyzed using the TapeStation.

## 3 Results

### 3.1 Optimization of RT-PCR detection assay for tomato mottle mosaic virus in tomato plants

**Figure 2:**
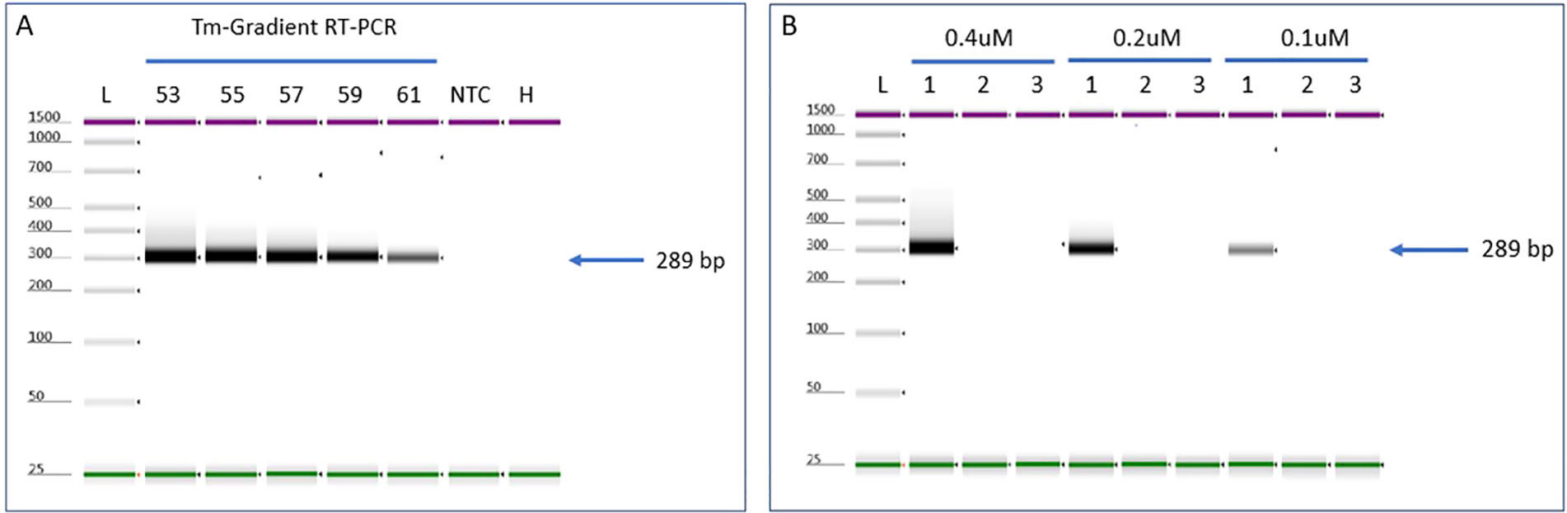
RT-PCR optimization parameters to detect ToMMV. **(A)** Evaluation of Tm and **(B)** Primer concentration for detecting ToMMV by using gradient RT-PCR. NTC, no template control; H, Healthy; L, Electronic Ladder.

Evaluation of temperature (Tm) gradient test for detection of ToMMV: Evaluation of *Tm* for the RT-PCR showed successful amplification in a range of annealing temperatures (53°C through 61°C) with amplicon products of 57.8 ng/µl at 53°C to 16.8 ng/µl at 61°C (**Figure 2**). A *Tm* of 55°C was selected to continue further verification assays, which is consistent with the original protocol published (Sui et al., 2017). We noticed that the RT-PCR amplicon intensity was higher at 53°C; however, nonspecific background noise was present (**Figure 2A** lane 1).

Evaluation of primer concentration test for detection of ToMMV: The evaluation of different primer concentrations for the RT-PCR showed successful amplification at a range of primer concentrations (0.1 µM, 0.2 µM, and 0.4 µM). The amplicon products ranged from 46 ng/µl at 0.4 µM, 27.4 ng/µl at 0.2 µM and 8.72 ng/µl at 0.1 µM (**Figure 2**). Based on these results, we selected 0.4 µM primer concentration for all our validation experiments (sensitivity, specificity, and selectivity). For this assay we used Tm 55°C, which we optimized (see above Tm section).

### 3.2 Sensitivity and specificity tests

**Figure 3:**
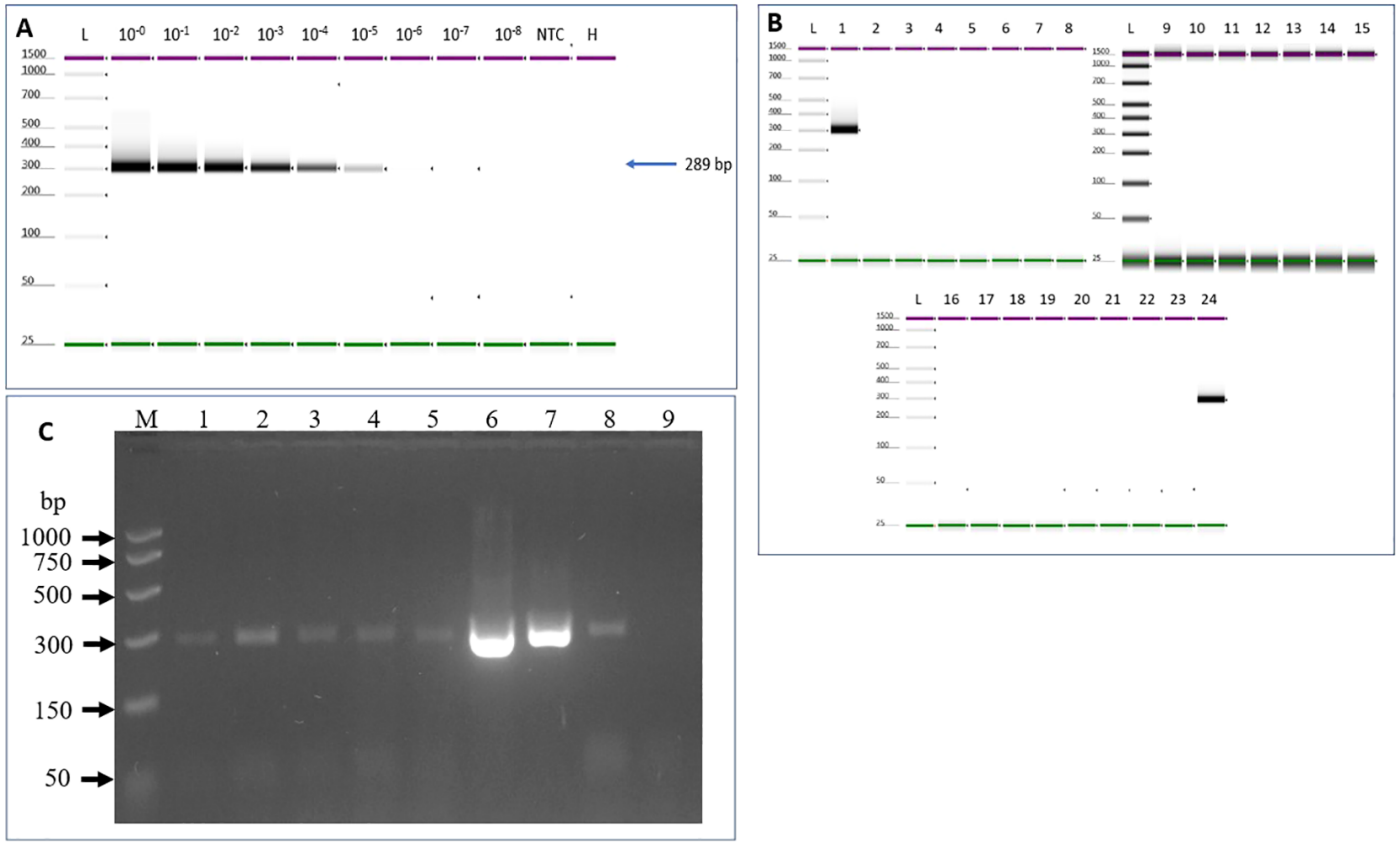
Sensitivity and specificity tests to determine ToMMV limit of detection. **(A)** Sensitivity was tested using 10-fold serial dilutions of total RNA of ToMMV-infected tomato plants spiked into healthy plant RNA. **(B)** Specificity of the assay was analyzed using targeted and non-targeted viruses and viroids. 1. ToMMV- (Tomato mottle mosaic virus), 2. TMV- (Tobacco mosaic virus), 3. ToMV- (Tomato mosaic virus), 4. ToBRFV- (Tomato brown rugose fruit virus-PV-1236), 5. TSWV- (Tomato spotted wilt orthotospovirus, 6. ToRSV- (Tomato ringspot virus), 7. ToCV- (Tomato chlorosis virus -PC-1242), 8. TICV- (Tomato infectious chlorosis virus -PV-1108), 9. PMMoV- (Pepper mild mottle virus-PV-0165) 10. BPeMV- (Bell pepper mottle virus -PC-0170), 11. WGMMV (Watermelon green mottle mosaic virus), 12. CGMMV- (Cucumber green mottle mosaic virus). 13. CEVd (Citrus exocortis viroid), 14. NTC (No template control), 15. Healthy control, 16. TCDVd (Tomato chlorotic dwarf viroid), 17. TASVd (Tomato apical stunt viroid), 18. MPVd (Mexican papita viroid), 19. CLVd (Columnea latent viroid), 20. TPMVd (Tomato planta macho viroid), 21. PSTVd (Potato spindle tuber viroid) 22. NTC (no template control), 23. Healthy, 24. positive (ToMMV) control, and L- (Electronic Ladder), NTC- (No template control), H- (Healthy). **(C)** Validation of the developed RT-PCR method for detection of a diverse collection of ToMMV isolates. Lane M: PCR marker (Promega); Lane 1: South Carolina isolate V13-478 (SC13-05); Lane 2: New York isolate 1 V13-481 (NY13-01) Lane 3: New York isolate 2 V13-481 (NY13-02); Lane 4: California isolate 1 V16-07 (CA16-01); Lane 5: California isolate 2 V16-08 (CA16-02); Lane 6: ToMMV Mexican isolate -MX5; Lane 7: Positive Control 1; Lane 8. Positive Control 2, Lane 9: Healthy plant control.

Evaluation of assay sensitivity was carried out based on the optimized assay conditions of 55°C and 0.4 µM primer concentration. The detection limit of the assay is 10^-5^ (0.25 pg/μL) dilution where an amplicon band of 289bp can be easily detected (**Figure 3A**). The developed assay was tested on naturally infected tomato plants that were grown in both field and greenhouse settings, as detailed in the materials and methods section. The RT-PCR results indicated that the protocol successfully detected all six ToMMV isolates (see **Figure 3C**).

Inclusivity was evaluated against several ToMMV isolates collected in the various States in the U.S., including ToMMV California isolate 1 V16-07 (CA16-01); California isolates 2 V16-08 (CA16-02); South Carolina isolate V13-478 (SC13-05); New York isolate 1 V13-481 (NY13-01); New York isolate 2 V13-482 (NY13-02); and from Mexico ToMMV-MX5. The RT-PCR result revealed all ToMMV isolates produced expected PCR products (**Figure 3C**).

Assay exclusivity was analyzed using eleven non-target viruses and seven viroids. Results through electrophoresis on an agarose gel revealed that the one-step RT-PCR with the ToMMV primers produced the expected size of amplicons from all six ToMMV-isolates, but not in any other non-target virus or viroid -infected samples, suggesting the assay is highly specific to ToMMV (**Figures 3B, C**).

### 3.3 Selectivity test

**Figure 4:**
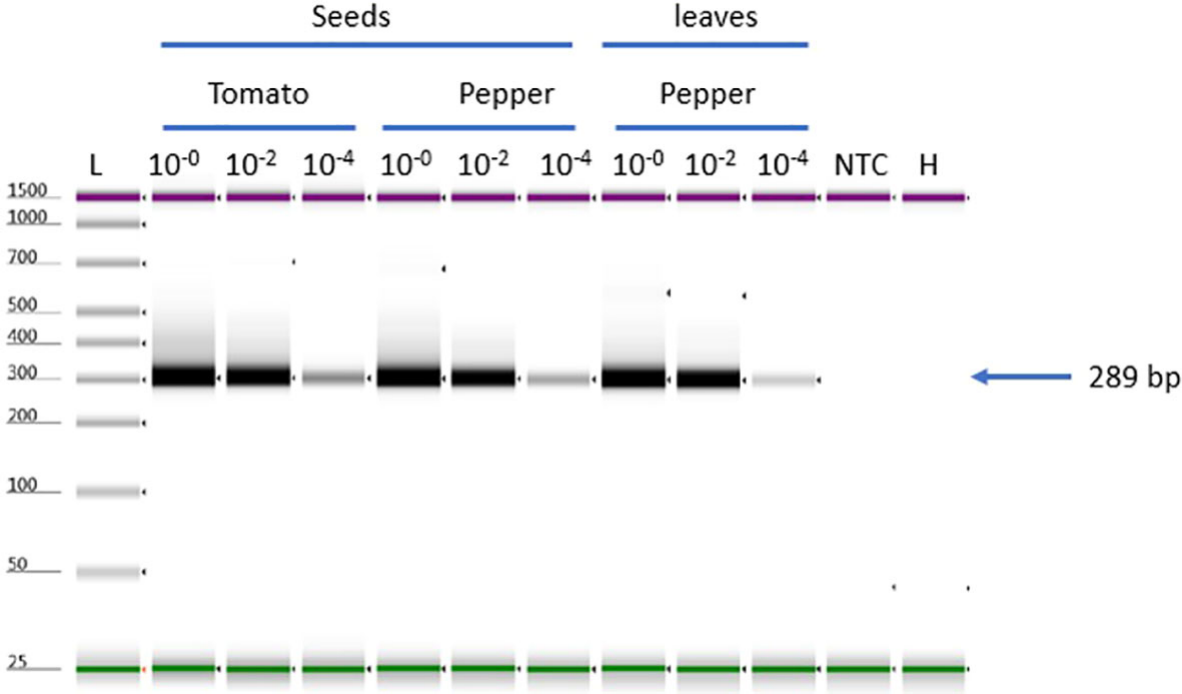
ToMMV-specific detection using different tissue matrices for selectivity test, Selectivity test to validate ToMMV (three matrices tomato -seeds, pepper -seeds and leaves). NTC, no template control; H, Healthy; L, Electronic Ladder.

Selectivity of the assay was assessed by spiking ToMMV RNA into RNA extracts from three different healthy plant matrixes including tomato (*Solanum lycopersicum*) seeds, Pepper (*Capsicum annum*) seeds and leaf tissue. Both tomato and pepper plant species have been reported as hosts of seed borne ToMMV. ToMMV-specific bands were detected in all matrices with three concentrations: high 10^-0^, medium (10^-2^), and low (10^-4^). The results showed that all matrices had a uniform detection efficiency (**Figure 4**); there is no effect between host and tissue type (seed or leaf matrix).

## 4 Discussion

Tomato mottle mosaic virus (ToMMV) is an economically important species of the genus Tobamovirus with impacts on the yield and quality of tomato fruits, pepper, and eggplant (Sui et al., 2017; Chai et al., 2018; Li et al., 2021 and Li et al., 2014) and extending to pea plants (*Pisum sativum*) (Zhang et al., 2022). Using some historical seed accessions, it was shown that ToMMV may have broader worldwide distribution and existence prior to its official description (Schoen et al., 2023). Realizing the importance of this emerging tobamovirus, it is important to have reliable and routine tools for ToMMV detection. Furthermore, under field conditions, mixed infection by closely related viruses with similar mottling and mosaic symptoms pose difficulty in disease diagnosis and the specific detection of the virus is necessary through sequence-based detection (Sui et al., 2017; Tettey et al., 2022; Luria et al., 2017; Fidan et al., 2021; Yan et al., 2021). In recent years, great efforts have been devoted in developing sensitive detection methods for ToMMV, including conventional RT-PCR (Sui et al., 2017; Tettey et al., 2022), real-time RT-PCR (Tiberini et al., 2022; Haghi et al., 2023), and reverse transcription loop-mediated isothermal amplification (RT-LAMP) (Kimura et al., 2023). However, it is important that these methods are vigorously validated and tested in different lab settings. In the present study, we focused on validating the conventional RT-PCR that was developed for specific detection and further identification of ToMMV (Sui et al., 2017) on seed and plant samples. The test can be extended to a sequence based-identification by conducting Sanger sequencing directly using the amplicon generated from the RT-PCR reaction. This sequence-based identification can be important to meet quarantine regulation test requirements in cases like first detections in a country, region, or an isolated environment.

The United States and other countries import tomato and pepper seeds from several Asian and European countries. To avoid seeds with ToMMV infection from countries where ToMMV is known to be present, we have validated an RT-PCR protocol that can specifically detect ToMMV in tomato and pepper. Since tomato-infecting tobamoviruses are closely related, sometimes ToMMV is misinterpreted due to its close sequence identity with other tobamoviruses. An important issue for detecting tomato-infecting tobamoviruses is that all the tomato-infecting tobamoviruses share serological reactions and thus cross react in the ELISA test (Turina et al., 2016; Sui et al., 2017), decreasing the utility of this assay. The best way to overcome this is to design and perform specific conventional RT-PCR methods followed by Sanger sequencing of amplicons. Towards that goal, Sui et al., 2017 developed a conventional RT-PCR to detect ToMMV and other two closely related viruses ToMV and TMV. From that RT-PCR assays, we have selected the ToMMV-primer for validation in the present study. We used standard parameters for method validation of the developed RT-PCR. We have evaluated different annealing temperatures, Tm- 53°C through 61°C and identified the highest amplification at 55 °C which is consistent with the protocol published (Sui et al., 2017). When *Tm* was reduced below 55°C the amplification amplicon intensity was higher but nonspecific background noise started appearing. It is quite common that a reduced *Tm* could increase nonspecific background. We also verified the primer concentration and identified 0.4 µM as optimal for higher amplicon production, which is similar to the Sui et al., 2017.

Sensitivity was tested using 10-fold serial dilutions of total plant RNA of ToMMV-RNA into healthy tomato plant RNA and identified the detection limit to be 10^-5^ dilution. Furthermore, we have evaluated the specificity of the assay using eleven non-target viruses and seven viroids. The selected viruses either belong to the same genus as ToMMV or commonly infect tomato and other plant species and we determined that this RT-PCR assay was specific to diverse ToMMV isolates. Sui et al., 2017 tested the specificity and found no cross-reactivity to non-targeted viruses such as PepMV, TSWV, TCSV, TYLCV, CGMMV, PMMoV, ToBRFV viruses and PSTVd viroid; we have added additional viruses in the specificity test and results are consistent with the Sui et al., 2017.

Selectivity of the assay was assessed by spiking ToMMV RNA into RNA extracts of healthy tomato (*Solanum lycopersicum*) seeds, pepper (*Capsicum annum*) seeds and leaf tissue matrices. Our results showed no difference between hosts, seeds, or leaf matrix. The original published protocol by Sui et al., 2017 did not perform a selectivity test; in this study the selectivity test did not show a negative effect of the matrix used in the performance of the assay. These results demonstrated that a single common protocol is effective for ToMMV detection across tissue or host types.

## 5 Conclusion

The validation results for ToMMV, which include performance measures of sensitivity, specificity, and selectivity measurements, along with samples collected from natural field conditions, demonstrate that the RT-PCR protocol is highly effective in specifically detecting ToMMV in both tomato and pepper plant tissues, as well as in their seed tissues. The protocol validated in this study is reliable for identifying the presence of the virus in these important agricultural crops.

## Data Availability

The raw data supporting the conclusions of this article will be made available by the authors, without undue reservation.
